# *Paeonia × suffruticosa* (Moutan Peony)—A Review of the Chemical Composition, Traditional and Professional Use in Medicine, Position in Cosmetics Industries, and Biotechnological Studies

**DOI:** 10.3390/plants11233379

**Published:** 2022-12-05

**Authors:** Halina Ekiert, Marta Klimek-Szczykutowicz, Agnieszka Szopa

**Affiliations:** 1 Chair and Department of Pharmaceutical Botany, Faculty of Pharmacy, Medical College, Jagiellonian University, Medyczna 9, 30-688 Kraków, Poland; 2 Department of Dermatology, Cosmetology and Aesthetic Surgery, The Institute of Medical Sciences, Medical College, Jan Kochanowski University, al. IX Wieków Kielc 19a, 25-516 Kielce, Poland

**Keywords:** tree peony, moutan, paeonol, paeoniflorin, therapeutic effects, cosmetic applications

## Abstract

The aim of this review is to perform a systematic review of scientific papers and an in-depth analysis of the latest research related to *Paeonia × suffruticosa* Andrews as a valuable plant species, important in pharmacy and cosmetology. *P. × suffruticosa* bark root-*Moutan cortex* is a medicinal raw material formerly known from traditional Chinese medicine (TCM) but less common in official European medicine. It was introduced for the first time in the European Pharmacopoeia Supplement 9.4 in 2018. In this work, the numerous possible applications of this raw material were depicted based on modern professional pharmacological studies documenting its very valuable medicinal values, including antioxidant, cytoprotective, anti-cancer, anti-inflammatory, cardioprotective, anti-atherosclerotic, anti-diabetic and hepatoprotective activities. The scientific studies indicated that the profile of raw material activity is mainly due to paeonol, paeoniflorin and 1,2,3,4,6-penta-O-galloyl-β-D-glucopyranose. Moreover, the significance of this plant (its different organs) in the production of cosmetics was underlined. *P. × suffruticosa* finds increasing application in cosmetology due to research on its chronic dermatitis, anti-aging and brightening effects. Furthermore, some biotechnological research has been described aimed at developing effective in vitro micropropagation protocols for *P. × suffruticosa.*

## 1. *Paeonia* Genus and *Paeonia lactiflora* and *Paeonia veitchii* as Known Medicinal Plants—General Characteristic

The classification of the genus *Paeonia* (Paeoniaceae) is complex from a taxonomic point of view. The species are divided according to three sections: Moutan DC., Paeon DC. and Onaepia Lindley [1,2]. The section on Moutan DC. contains the evolutionarily older shrub peonies. The Moutan section has two subsections: subsect. Vagintae and subsect. Delavayanae including peony species, such as *P. cathayana, P. decomposita, P. jishanensis, P. ostii, P. qiui, P. rockii, P. rotundiloba, P. delavayi, P. ludlowii* and *P. suffruticosa* [1,3]. Paeon DC. is an extensive section consisting of 26 varieties of herbaceous plants with fleshy leaves with deep indentations. Characteristic species here include *P. lactiflora* and *P. veitchii* [1]. In the section Onaepia Lindley, there are several species of peonies with grassy leaves, including *P. brownii* and *P. californica* [1].

The Latin name of the genus, *‘Paeonia’*, is derived from Greek legend about Paeon and Pluto. Paeon was a disciple of Aesculapius, the Greek god of medicine. According to the legend, Paeon used a peony concoction to heal Pluto, who had been wounded in the Trojan War. Aesculapius, jealous of his student’s healing skills, plotted to kill Paeon. Pluto discovered it and thwarted the plot by transforming Paeon into a peony [4].

Peonies are native to Asia, Europe and North America. The section Moutan DC., which contains all woody species, is restricted in the wild to Central and Southern China, including Tibet. The section Onaepia is present in the west of North America, and the section Paeonia occurs in a band stretching roughly from Morocco and Spain to Japan [5].

Today, peonies are very popular plants as ornamental species. More than 1200 varieties of Chinese and tree peonies have already been bred, mainly in China and Japan, but also in other geographical areas, including France, England and the United States. In Europe and North America, peonies are planted in gardens and parks for their showy flowers of great aromatic and ornamental value; the flowers range in form and color from snow-white, light and dark pink, to bright red and burgundy [6].

The known pharmacopoeial raw materials of the genus *Paeonia* (Peony) are the roots extracted from two species: *Paeonia lactiflora* Pall. and *P. veitchii* Lynch. The monographs *Paeoniae radix rubra* (peony red root) and *Paeoniae radix alba* (peony white root) are listed in the 10th edition of the European Pharmacopoeia [7]. These raw materials are also listed in the modern Chinese Pharmacopoeia [8] and are accepted by the Committee on Herbal Medicinal Products (HMPC) [9]. *Paeoniae radix rubra* is extracted from the species *Paeonia lactiflora* and *P. veitchii*; it is the whole root dried in the sun with the reddish, thick outer bark and with only the rhizome and rootlets removed. *Paeoniae radix alba* can only be obtained from *P. lactiflora*; the bark is removed from the root and the exposed powdery-white layer is the raw material subjected to boiling and then drying [10]. According to the requirements of the European Pharmacopoeia, raw materials are to be standardized for paeoniflorin content. The red root should contain a min. 1.8% of this compound, while white 1.6% [11]. *Paeoniae radix rubra* is mostly used to treat hematemesis, warm toxin, amenorrhea, dysmenorrhea, blood stasis, abdominal pain, red eyes, headache and carbuncles. *Paeonia radix white* has a biological effect such as to treat irregular menstruation and to protect the liver [12,13,14].

Both *P. lactiflora* and *P. veitchii* are species that have their natural habitats in Asia (mainly in China and Siberia). *P. lactiflora* Pall. (Milk Quince Peony) is popular in ornamental horticulture. There are 5000 varieties in the world. In Ukraine, cultivars grown as *P. lactiflora* are perennials and may differ in size and shape, color, color and flowering time. Hybrids and varieties of *P. lactiflora* are grown in gardens and orchards and are used in folk medicine [15,16,17]. Interestingly, these raw materials in Traditional Chinese Medicine (TCM) have quite different medicinal uses. Red root, according to TCM, is administered for febrile conditions, to reduce swelling and for menstrual disorders [18]. White root is used as an analgesic, sedative and anti-inflammatory agent. It is also found in the composition of herbal mixtures used to treat depressive disorders. However, modern pharmacological research indicates that both raw materials have similar activities, acting as a: sedative, anti-inflammatory, hypotensive, analgesic, relieving convulsions and inhibiting platelet agglutination agent [19,20,21,22].

## 2. *Paeonia × suffruticosa* as a New Pharmacopoeial Plant Species

A new raw material of the genus Paeonia, whose monograph first appeared in Supplement 9.4 to the 9th edition of European Pharmacopoeia [23] in 2018, is the root bark of *Paeonia × suffruticosa* Andrews-*Moutan cortex*. Monographs of *P. × suffruticosa* are also listed in the: Chinese [8], Japanese [24], Korean [25] and Vietnamese Pharmacopoeias [8], and, invariably, in the latest (10th) edition of the European Pharmacopoeia [7]. The pharmaceutical raw material is the bark of the root *Paeoniae × suffruticosa cortex radicis-Moutan cortex*, collected in autumn, dried, whole or broken, rubbed or not. The Chinese Pharmacopoeia distinguished the two types of *P. × suffruticosa* barks: *Liandapi* (the root is harvested in autumn, removed from the roots and soil, torn off the root bark and dried in the sun) and *Guadanpi* (coarse bark removed from the lignified parts and dried in the sun) [26]. *Moutan cortex*, according to the requirements of the European Pharmacopoeia 10th ed., should contain min. 2.2% paeonol and a minimum of 1.1% paeoniflorin [7].

TCM attributes *Moutan cortex* with the following effects: antipyretic, regulating menstrual disorders, accelerating the healing of ulcers, improving blood circulation and reducing swelling. In the treatment of fever, the raw material of peony root bark is administered in its raw form, while its alcoholic solutions are used to improve circulation and remove stasis. This raw material should not be used in pregnant women and those with heavy menstruation [26].

The currently known pharmacological potential of *P. × suffruticosa* is undoubtedly determined by its rich chemical composition. The most important groups of secondary metabolites are phenolic compounds and monoterpenoid glycosides [6,27,28,29,30,31,32,33]. This species also includes triterpenoid saponins, flavonoids, phenolic acids and polysaccharides [6,32,34]. The most important compounds responsible for the valuable biological activity of the raw material are paeonol (phenolic compound) and paeoniflorin (monoterpenoid glycoside), and partly, also 1,2,3,4,6-penta-O-galloyl-β-D-glucopyranose [6].

Currently, valuable scientific publications focus on the increasing pharmacological action of the extract, mainly from *Moutan cortex*, as well as the compounds present in it. They include research on, among others, antioxidant, anti-inflammatory, cytoprotective, anti-cancer and neuroprotective activities [19,20,35,36,37,38,39,40,41,42,43,44,45,46,47,48,49,50,51,52,53,54,55,56,57,58,59,60,61,62,63,64,65,66,67,68,69]. Other studies also include activities which are important due to modern civilization’s diseases, such as cardioprotective and anti-atherosclerotic, anti-diabetic and hepatoprotective effects [21,34,70,71,72,73,74,75,76,77,78,79,80,81,82,83,84,85].

*P. × suffruticosa* bark root extracts also have scientifically proven cosmetic properties, such as: antioxidant, anti-aging and skin brightening [86,87]. Recent reports revealed paeonol from *P. × suffruticosa* exhibited good effects on chronic dermatitis, such as atopic dermatitis (AD) and psoriasis [88].

The aim of this work is to deeply characterize the species, with a particular focus on biological activity, with potential applications in medicine, pharmacy and cosmetology based on scientific reports.

## 3. *Paeonia × suffruticosa*—General Characteristics

*Paeonia × suffruticosa* Andrews belongs to the Paeoniaceae family [89]. Throughout the world, it is known as: moutan, moutan peony, tree peony (English), Strauch-Pfingstrose (German), Mudan (Chinese) and moran (Korean) [2]. *P. × suffruticosa* is a plant known by numerous Latin synonymous names, such as: *P. × arborea* C.C.Gmel., *P.× chinensis* Oken, *P. × fruticosa* Dum. Cours., *P. × moutan* Sims, *P. × moutan* var. *anneslei* Sabine, *P. × moutan* var. *papaveracea* (Andrews) DC., *P. × papaveracea* Andrews, *P. × suffruticosa* f. *anneslei* (Sabine) Rehder, *P. × suffruticosa* var. *banksii* (Sabinene) L.H. Bailey, *P. × suffruticosa* var. *humei* (Sabinene) L.H. Bailey, *P. × suffruticosa* f. *maculata* Hong C. Zheng, *P. × suffruticosa* var. *papaveracea* (Andrews) Kern., *P. × suffruticosa* var. *purpurea* Andrews, *P. × suffruticosa* f. *rubida* Hong C. Zheng and *P. × yunnanensis* W.P. Fang [2].

The word “moutan” is of Chinese origin, and means “chat” or “short conversation”. It was first used in the name of the raw material in 1808 and was fixed in botanical nomenclature in the 2nd edition of the English *Hortus Kewensis* [89,90]. *P. × suffruticosa*, in Chinese colloquial language, is also called “mudan”, and is just as famous for its ornamental as well as its medicinal uses: its flower is a symbol of elegance and prosperity, while its bark root, namely “mudanpi” in Chinese, is broadly used in TCM as an adjuvant for cardiovascular and gynecological diseases [91,92,93].

Botanically and genetically, *P. × suffruticosa* is a very interesting and not fully understood plant. In 2001, the contemporary British taxonomist S. G. Haw [89] described *P. × suffruticosa* as a hybrid, but this was not supported by any evidence. Genetic analysis has shown that the five species from the subsection together constitute the origin of the tree peony varieties that arose before World War II. *P. cathayana* is indicated as the primary maternal species due to studies in which three-quarters of the fifty subjects have the same chloroplast DNA as this species. The remaining cultivars have chloroplast DNA identical to *P. qiui*, rarely from *P. ostii* and partly from *P. rotundiloba*. However, in nuclear DNA, homology with *P. rockii* is greatest, with *P. qiui*, *P. ostii*, *P. cathayana* and *P. jishanensis* to a lesser extent [89,94].

*P. × suffruticosa* is a shrubby plant reaching from 1 up to 4 m in height. The proximal leaves are doubly tripartite, and the terminal leaves are divided into three lobes, which divide into another two to three lobes, each ending in a sharp apex. The leaves are ovate or longitudinally ovate in shape, measuring 4.5–8 by 2.5–7 cm. Both surfaces of the leaf blade are smooth. Flowers are large, single (in cultivated varieties sometimes double), 10–17 cm wide and set directly on the stem. The flower has five green, broadly ovate and irregular calyx sepals each. The petals of the flower corolla are inversely ovate in shape and measure 5–8 by 4.2–6 cm. In single flowers, petals occur in number from 5 to 11; they are white, pink, red or reddish-purple in color. The circular flower base is purple in color. The plant blooms from April to May. It bears fruit in August [89]. The root extends over 1 m into the ground and is 5–12 mm in diameter and 1–4 mm thick. The outer surface is grayish-brown or yellowish-brown, with numerous transverse protrusions; it is pink when the bark falls off. The inside is pale grayish-yellow or pale brown, with distinct fine longitudinal stripes, usually with pale crystals [26].

## 4. *Paeonia × suffruticosa*—Phytochemical Characteristics

To date, about 119 compounds have been isolated and identified from the bark of *P. × suffruticosa root—Moutan cortex*, with phenolic compounds and monoterpenoid glycosides being the predominant compounds among them (Table 1) [29,94,95,96,97,98,99,100,101].

**Figure 1:**
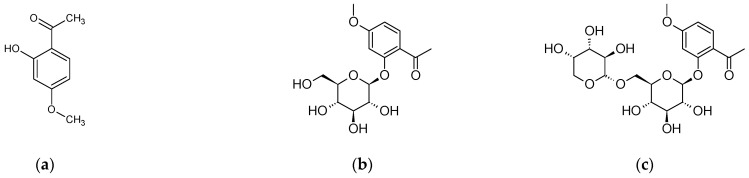

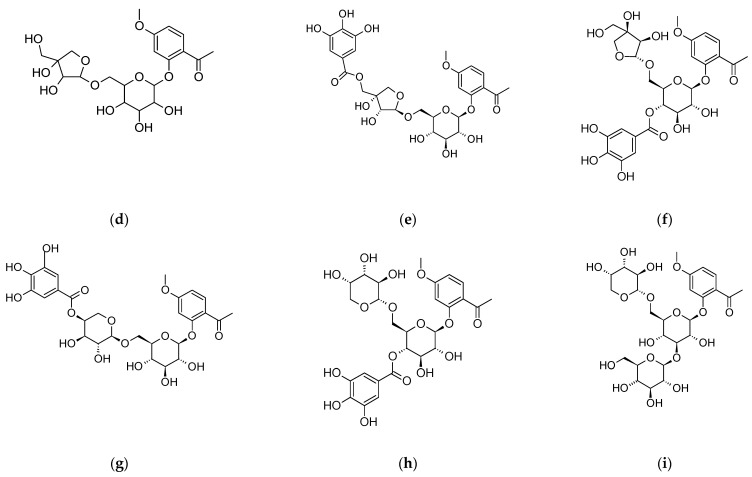
Chemical structure of: (**a**) paeonol, (**b**). paeonoside, (**c**) paeonolide, (**d**) apiopaeonoside, (**e**) suffruticoside A, (**f**) suffruticoside B, (**g**) suffruticoside C, (**h**) suffruticoside D, (**i**) suffruticoside E.

**Figure 2:**
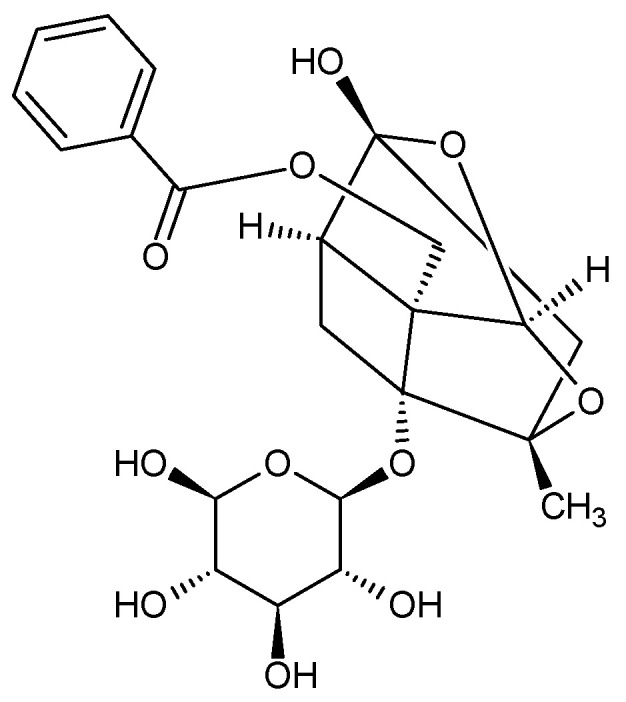
Chemical structure of paeoniflorin.

The main and characteristic compound of the raw material is the phenolic compound paeonol (2′-hydroxy-4′-methoxyacetophenone, peonol) and its glycosides, such as: paeonoside, paeonolide, apiopaeonoside and suffruticosides A-E (Figure 1). Among the monoterpenoid glycosides in the species *P. × suffruticosa*, several pairs of isomers have been identified, including α- and β-benzoylpaeoniflorin and benzoylpaeoniflorin and paeonioside A. The common compounds in all species of the genus *Paeonia* include terpene glycoside paeoniflorin (Figure 2) and its analogues (oxypaeoniflorin, albiflorin, galloylpaeoniflorin, galloyloxypaeoniflorin, benzoylpaeoniflorin, mudanpioside A-J, α-benzoyloxypaeoniflorin, β-benzoyloxypaeoniflorin, 6-O-vanillyoxypaeoniflorin, suffrupaeonidanin A-F, paeonidanin A and C, suffruyabiosides A and B, paeoniside A and B, suffrupaeoniflorin A and B, oxypaeonidanin, 9-epi-oxypaeonidanin, 8-O-benzoylpaeonidanin, 9-O-butyloxypaeonidanin, 9-O-butylpaeonidanin, *p*-hydroxylbenzoylpaeonidanin, 4-O-methylsuffrupaeoniflorin B, paeoniflorin-4-ethyl ether, 4-O-butylpaeoniflorin, 4-O-methylmoudanpioside C, 4-O-methylbenzoyloxypaeoniflorin, oxypaeoniflorin sulfonate, 4-O-methyloxypaeoniflorin, 4-O-methylgalloyloxypaeoniflorin, 4-O-butyloxypaeoniflorin, 4-O-methylpaeoniflorin, 8-O-debenzoylpaeoniflorin, 4-O-methylbenzoylpaeoniflorin) with a specific structure, the so-called “cage-like pinnae skeleton” [102].

**Figure 3:**
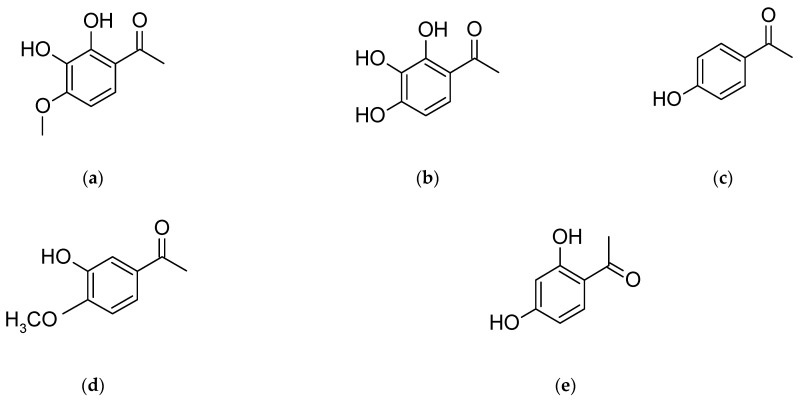
Chemical structure of (**a**) 2,3-dihydroxy-4-methoxyacetophenone, (**b**) gallacetophenone, (**c**) *p*-hydroxyacetophenone, (**d**) 3-hydroxy-4-methoxyacetophenone, (**e**) reacetophenone.

**Figure 4:**
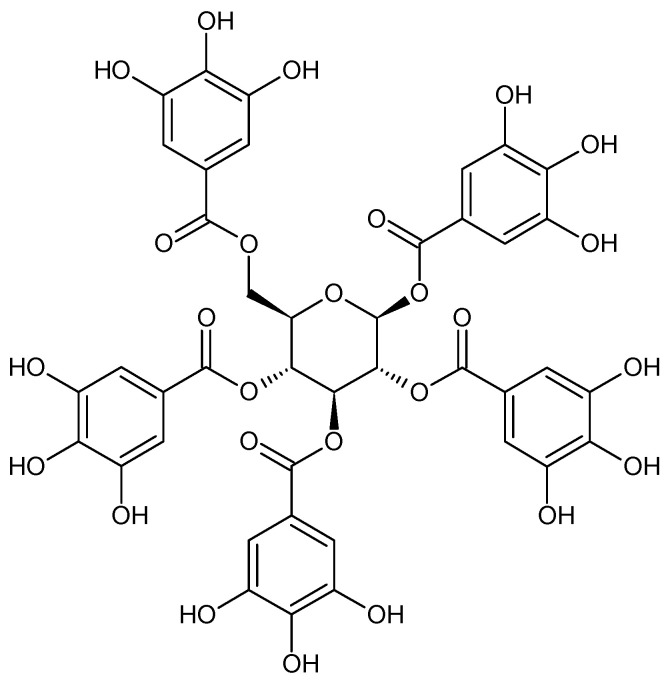
Chemical structure of 1,2,3,4,6-penta-O-galloilo-β-D- glucopyranose.

Acetophenones are also characteristic compounds of *P. × suffruticosa*, such as: 2,3-dihydroxy-4-methoxyacetophenone, gallacetophenone, *p*-hydroxyacetophenone, 3-hydroxy-4-methoxyacetophenone and reacetophenone (Figure 3). Noteworthy compounds also include peonisotujone and peonisuffrone (a tricyclic compound). Peonisotujone is the first identified compound of natural origin with an *ortho*-menthane-type monoterpenoid structure having a cyclopropane ring [31]. Additionally the presence of a specific structure, 1,2,3,4,6-penta-O-galloyl-β-D-glucopyranose (Figure 4), which plays a significant role determining the biological activity of the raw material, was found in the root [27,28,103].

**Table 1:** The most important chemical ingredients of *Paeonia × suffruticosa*.

Group of Compounds	Compounds	References
**Terpenoid compounds**	Monoterpenenoids deoxypaeonisuffrone, isopaeonisuffral, 6-methoxy-paeoniflorigenone, 3-O–methylpaeonisuffral, paeonifloringenone, paeoniflorin A, paeonisothujone, paeonisuffral, paeonisuffrone, (-)-paeonisuffrone Monoterpenenoid glycosides: benzoylpaeoniflorin, α-benzoyloxypaeoniflorin, β-benzoyloxypaeoniflorin, 8-O-benzoylpaeonidanin, 9-O-butyloxypaeonidanin, 4-O-butyloxypaeoniflorin, 9-O-butylpaeonidanin, 4-O-butylpaeoniflorin, 8-O-debenzoylpaeoniflorin, galloyloxypaeoniflorin, galloylpaeoniflorin, *p*-hydroxylbenzoylpaeonidanin, 4-methylbenzoyloxypaeoniflorin, 4-methylgalloyloxypaeoniflorin, 4-methylmoudanpioside C, 4-methyloxypaeoniflorin, 4-methylsuffrupaeoniflorin B, 4-O-methylpaeoniflorin, 4-O-methylbenzoylpaeoniflorin, mudanpioside A-J, oxypaeonidanin, 9-epi- oxypaeonidanin, oxypaeoniflorin sulfonate, paeonidanin A and C, paeoniflorin, paeoniflorin B, paeoniflorin-4-ethyl ether, paeoniside A and B, suffrupaeonidanin A-F, suffrupaeoniflorin A and B, suffruyabiosides A and B, 6-O-vanillyoxypaeoniflorin Triterpenoids: betulinic acid, mudanpinoic acid A, oleanolic acid, palbinone, ursolic acid	[6,27,28,29,30,31,32]
**Triterpenoid saponins**	hederagenin, 30-norhederagenin	[6]
**Acetophenons**	2,3-dihydroxy-4-methoxyacetophenone, gallacetophenone, *p*-hydroxyacetophenone, 3-hydroxy-4-methoxyacetophenone, reacetophenone	[33]
**Phenols and their derivatives**	apiopaeonoside, galusan metylu, iriflophenone 2-O-β-D-glucopyranoside, methyl 3-hydroxy-4-methoxybenzoate, mudanoside C, paeonol, paeonolide, paeoniside, phenol, suffruticosides A–E	[6,33]
**Flavonoids**	apigenin 7-O-rhamnoside, isorhamnetin 3,7-di-O-glucoside, quercetin, kaempferol, kaempferol 3,7-di-O-glucoside, quercetin 3-O-galloylglucoside	[6,32]
**Phenolic and cyclohexanecarboxylic acids**	gallic acid, galloylquinic acid, *trans*-caffeic acid stearyl ester, quinic acid, *p*-hydroxybenzoic acid, 3-hydroxy-4-methoxybenzoic acid	[32]
**Resveratrol oligomers**	suffruticosol A, suffruticosol B, suffruticosol C, *trans*-gnetin H, *trans*-suffruticosol D, *trans*-ε-viniferin	[63]
**Catechins**	catechin, (+)-catechin-7-O-glucopyranoside, dimeric proanthocyanidin epicatechin-(4β-8)-catechin, epicatechin-3-O-gallate, flavan-3-ols catechin, epigallocatechin gallate	[104]
**Sterols**	campesterol, daucosterol, β-sitosterol	[6,31]
**Polysaccharides**	polisacharyd-2b	[34]
**Others**	adenosine, ainsliaside E, 1,2,3,4,6-penta-O-galloyl-β-D-glucopyranose, paesuffrioside, trigalloilo-glucose, thymidine, 1-tryptophan, uridine	[30]

In extracts of flowers’ characteristic compounds are terpenes, alcohols, alkanes, alkenes and alkatriens. The main monoterpenes are (Z)-β-ocimene, alloocimene, citronellol, citronellyl acetate, α-pinene and linalool [105,106]. In addition to the above-mentioned compounds, the presence of monoterpene glycosides, phenols, phenolic acids, tannins, flavonoids, irydoid glycosides, amino acids and sugars was confirmed in ethanol extracts from flowers [107]. The chemical composition of *P. × suffruticosa* flowers was presented in Table 2.

Seed oil of *P. × suffruticosa* contains mainly linolenic acid, linoleic acid, oleic acid and palmitic acid. Linolenic acid and linoleic acid together accounted for more than 60%, indicating that *P. × suffruticosa* seed oil can serve as a good dietary source of polyunsaturated fatty acid. On the contrary, arachidic acid, palmitoleic acid and gadoleic acid were present in smaller amounts. *P. × suffruticosa* seed oil also contains tocopherols (α-tocopherol, γ-tocopherol, δ-tocopherol), sterols (campesterol, stigmasterol, β-sitosterol, Δ-5-avenasterol, brassicasterol, fucosterol) and squalene [108].

## 5. *Paeonia × suffruticosa*-Studies on Biological Activity

### 5.1. Antioxidant Effect

Phenolic compounds found in *Moutan cortex* are mainly responsible for the antioxidant activity of the raw material.

Ethanolic extract of *Moutan cortex* at a concentration of 1 mg/mL reduces the production of reactive oxygen species (ROS) and oxidative stress-induced cytotoxicity in PC12 cells (rat adrenal pheochromocytoma cells) by enhancing the expression of genes for, among others, catechol-O-methyltransferase and hemoxygenase, which are involved in the regulation of cell cycle and free radical production [45].

The antioxidant effect was also confirmed in studies on mice exposed to cigarette smoke for 4 weeks, which caused inflammatory infiltration of the lungs, increased permeability of pulmonary vessels and increased levels of chemokines, cytokines and 4-hydroxynonenal (a biomarker of oxidative stress) in the lungs. Chronic treatment with peonol suppressed the aforementioned symptoms. In addition, extended studies on human bronchial epithelial cells showed that paeonol treatment reduced extracellular and intracellular ROS levels, inhibited mitogen-activated kinase (MAPK/NF-κB) signaling and reduced interleukin-8 (IL-8) levels induced by cigarette smoke extract [56].

In another study, a beneficial combined effect of paeonol (at a dose of 80 mg/kg) and 3-(3,4-dihydroxyphenyl)-2-hydroxypropionic acid (danshensu, the main component of *Salviae milthiorrhizae* radix at a dose of 160 mg/kg) was demonstrated in cases of isoproterenol-induced rat myocardial infarction (85 mg/kg). The authors of the study concluded that the mechanism of this effect may be related to the enhancement of antioxidant activity through activation of Nrf2 transcription factor signaling that controls the expression of genes encoding enzymes and cytoprotective proteins [64] (Table 3).

### 5.2. Cytoprotective Effect

Scientific studies prove the cytoprotective effect of paeoniflorin isolated from the *Moutan cortex*. Paeoniflorin (50–200 µg/mL) protected thymocytes (“pre-T lymphocytes”) from ^60^Co radiation-induced oxidative damage [38]. Another study showed that peoniflorin protected human cell lines (EA.hy926) from gamma radiation-induced oxidative damage through the Nrf2/HO transcription factor pathway [39]. In addition, some studies also suggest that paeoniflorin protects retinal pigment epithelial cells from oxidative stress by reducing ROS production and inhibiting activation of the caspase-3 pathway [40].

Moreover, studies conducted on different concentrations (1, 5, 10 or 20 μM) of galloylopaeoniflorin (a derivative of paeoniflorin) showed cytoprotective activity at 20 μM against hydrogen peroxide-induced cell damage and death in human keratinocytes HaCaT [41] (Table 3).

### 5.3. Anti-Inflammatory Effect

Studies conducted both in vitro and in vivo on the anti-inflammatory activity of *Moutan cortex* indicated that two compounds—paeonol and paeoniflorin—are mainly responsible for this effect.

Studies conducted with *Moutan cortex* extracts at concentrations of 0.1 and 0.3 mg/mL on regulatory mechanisms of cytokine and nitric oxide production, involved in immune activity of mouse macrophage/monocyte RAW264.7 cells, showed inhibition of nitric oxide synthase (iNOS) and inducible cyclooxygenase (COX-2) expression by suppressing phosphorylation of the inhibitory protein (I-κBα) transcription factor NF-κB [20].

Another study focused on gene expression changes in cultures of human gingival fibroblasts stimulated by lipopolysaccharides (LPS). The results suggested that a crude extract containing paeonol and paeoniflorin blocked the induction of inflammation by comprehensively inhibiting the activation of multiple genes associated with the formation of inflammation [65].

It was also demonstrated that paeoniflorin added at various concentrations (1, 10 or 100 µmol/L) 2 h before exposure to 10 mg/L lysophosphatidylcholine (LPC) for 24 h inhibits the production of inflammatory factors in LPC-induced human umbilical vein endothelial cells by inhibiting the HMGB1-RAGE/TLR-2/TLR-4-NF-κB pathway [66].

Studies of paeonol, paeoniflorin and 1,2,3,4,6-penta-O-galloyl-β-D-glucopyranose confirmed their inhibitory effects on tumor necrosis factor (TNF-α) synthesis and interleukin-6 (IL-6) production in synoviocytes (synovial membrane cells of the joint capsule) treated with pro-inflammatory agents in a dose-dependent manner [67].

Studies have shown that a methanolic extract of *Moutan cortex*, particularly 1,2,3,4,6-penta-O-galloyl-β-D-glucopyranose, inhibits IL-8 and monocyte chemotactic protein secretion in human monocytic cells (U937) stimulated with phorbol myristate acetate [68].

The anti-inflammatory effect of paeonol (at doses 150, 200 or 250 mg/kg) was also demonstrated by inhibiting inflammatory cytokines in lipopolysaccharide (LPS)-induced macrophage cells in mice. In addition, paeonol protected mice from lethal endotoxic shock. In vitro studies showed that paeonol regulated the production of TNF-α and the interleukins IL-1β, IL-6 and IL-10 through inactivation of I-κBα (NF-κB inhibitory protein), extracellular signal-regulated kinase (ERK1/2), c-JUN N-terminal kinase (JNK) and mitogen-activated protein kinase (p38 MAPK) [69].

Treatment with paeonol significantly improved the survival rate and mean arterial pressure (MAP) and attenuated the pathological damage to the lung tissue in acute lung injury rats. Western blotting revealed that paeonol also inhibited the total expression of HMGB1, NF-κB P65 and TNF-α in the lung tissue of acute lung injury rats. Moreover, paeonol increased the expression of HMGB1 in the nucleus, inhibited the production of HMGB1 in the cytoplasm and decreased the expression of P65 both in the nucleus and cytoplasm of lung tissue cells in LPS-induced acute lung injury rats. These findings indicate that paeonol may be a potential treatment for acute lung injury through its repression of the HMGB1-NF-κB P65 signaling pathway [35].

Examination of paeoniflorin on the activity of M1 pro-inflammatory and M2 anti-inflammatory macrophages showed that the compound can suppress the activity of M1 cells while increasing the function of M2 cells. This action can be used to treat autoimmune and autoinflammatory diseases [36].

The studies on chondrogenic cell line ATDC5 cells cultured with IL-1b showed that peonol from *P. × suffruticosa* inhibits numerous factors of osteoarthritis, including expressions of IL-6, TNF-α, NADPH oxidase 2 (NOX2), prostaglandin–endoperoxide synthase 2 (PTGS2), nucleobindin-2 (NUCB2)/nesfatin- 1, intercellular adhesion molecule 1 (ICAM-1), vascular cell adhesion molecule 1 (VCAM-1), matrix metalloproteinase-3/13 (MMP-3/13) and degradation of type II collagen [37] (Table 3).

### 5.4. Anticancer Effect

Many experiments on the anticancer properties of the raw material have been based on studies of the isolated compounds, paeonol and paeoniflorin. It was demonstrated that paeonol reduces paclitaxel resistance in human breast cancer cells by regulating the expression of transgelin 2 [42] and also exerts an anti-cancer effect on colon cancer cells by suppressing prostaglandin (PGE-2) synthesis and COX-2 expression. In addition, paeonol inhibits metastasis of melanoma [43] and chondrosarcoma [44] and, at doses of 150 and 300 mg/kg, induces apoptosis of EMT6 breast cancer cells [46] at concentrations (7.81–250 mg/L) of human HepG2 liver cancer cells [47] and at doses of 100, 200 or 400 mg/kg/day of HepA cells in a mouse model [48]. 

In studies on mouse forestomach carcinoma (MFC) cell lines and on SGC-7901 human gastric cancer cells, paeonol was shown to cause dose-dependent inhibition of cell proliferation and induction of apoptosis. Cell cycle analysis showed reduced cell proliferation in G/G1 phase, with arrest in S phase. In MFC and SGC-790 cells, paeonol significantly decreased the expression of proteins that regulate the release of cytochrome *c* from mitochondria (Bcl-2) and increased the expression of apoptosis-accelerating protein (Bax) in a concentration-dependent manner. Administration of paeonol to mice with an MFC tumor significantly reduced tumor growth and caused its regression [49].

Studies demonstrated that paeoniflorin inhibits the proliferation and invasion of breast cancer cells by suppressing the signaling pathway for the gene encoding the Notch-1 single-pass trans-membrane receptor [109] and inhibits the macrophage-dependent metastasis of lung cancer [50]. In addition, paeoniflorin at concentrations of 10 and 20 μM inhibits proliferation and induces apoptosis of human glioma cells through up-regulation of microRNA-16 and down-regulation of metalloproteinase-9 [51].

In vitro studies of 1,2,3,4,6-penta-O-galloyl-β-D-glucopyranose isolated from *Moutan cortex* showed its antiproliferative activity on human hepatocellular carcinoma cell line SK-HEP-1 [52].

Scientific studies further showed that *Moutan cortex* extracts show greater selectivity in inhibiting growth against bladder cancer cells than mitomycin C, doxorubicin or cisplatin. The raw material also reduced the expression of angiogenesis-stimulating factors, including vascular endothelial growth factor (VEGF) [53].

Aqueous extracts of *P. × suffruticosa* were tested for action on pancreatic cancer cell line PANC1 and in vivo in mouse xenograft tumors. The extracts induced stress on endoplasmic reticulum (ER)-related proteostasis and affected mitochondrial membrane potential to increase autophagosome numbers and block their degradation, followed by autophagy induction and, finally, cell apoptosis. Nevertheless, oral administration of *P. × suffruticosa* aqueous extracts, alone or in combination with gemcitabine in mice, delayed tumor growth in a xenograft model without affecting body weight [54].

Another study demonstrated that paeonol can exert antitumor effects on hepatocellular carcinoma (HCC) cells by targeting survival via the COX-2/PGE2 signaling pathway. Peonol significantly inhibited the proliferation of human liver cancer cell line (HepG2) and human hepatocarcinoma cell line (SMMC-7721) and induced apoptosis, concomitant with the down-regulation of survival. The levels of COX-2 and PGE2 were also reduced by paeonol [55] (Table 3).

### 5.5. Cardioprotective and Anti-Atherosclerotic Effects

Scientific studies of isolated compounds present in both *Paeoniae alba radix* and *Moutan cortex* for their anti-aggregation and anti-coagulation properties showed that paeonol, paeoniflorin, benzoylpaeoniflorin and benzoyloxypaeoniflorin are the main compounds that together can contribute to improving blood circulation. These compounds had an inhibitory effect on thrombocyte aggregation and blood coagulation. In addition, it is possible that other compounds in the raw materials, i.e., methyl gallate, (+)-catechin, paeoniflorigenone, galloylpaeoniflorin and daucosterol may also be involved in improving circulation [21].

Another study showed that *Moutan cortex* extract administered at a dose of 1.98 g/kg for 14 days exerts a protective effect in a rat model of ischemia and reperfusion [78].

Studies of paeoniflorin administered at doses of 5, 10 and 20 mg/kg confirmed its ability to alleviate acute myocardial infarction in rats by inhibiting inflammatory processes and nitric oxide synthase (iNOS) signaling pathways [79]. It was also demonstrated that paeoniflorin reduces vascular damage and the expression of E-selectin and the intercellular adhesion molecule (ICAM-1) in a mouse model of cutaneous Arthus reaction [80]. Paeonol and paeoniflorin enhance thrombus recanalization by inducing endothelial growth factor-165 [81] and up-regulating urokinase-type plasminogen activator, respectively [82]. In both cases, the mechanism of action was related to the mitogen-activated kinase (MAPK) signaling pathway.

It was also confirmed that paeonol prevents the development of atherosclerosis by inhibiting monocyte adhesion, induced by the oxidized form of low-density lipoprotein (LDL), to the vascular endothelium through inhibition of the mitogen-activated kinase (MAPK) signaling pathway [83].

Studies have shown that the paeoniflorin bioactive compound from *P. × suffruticosa* prevented arterial thrombosis in vivo from the dose of 10 mg/kg without prolonging bleeding time or blood clotting time in rats [84] (Table 3).

### 5.6. Antidiabetic Effect

In in vitro studies conducted on four models (intestinal brush border epithelial cells-BBMV, cells of the H4IIE (rat hepatoma) line, human fibroblast cells-Hs68 and mouse adipocytes 3T3-L1), it was shown that *Moutan cortex* extract and its main component, paeonol, have antidiabetic effects by inhibiting glucose uptake by intestinal brush border membrane vesicles (BBMV) and increasing glucose uptake in human skin fibroblast cells (Hs68) and mouse adipocytes (3T3-L1). Paeonol (at doses of 200 and 400 mg/kg) was also shown to improve glucose tolerance in an in vivo model [85].

In a study of encephalopathy in rats with streptozocin-induced diabetes, a significant decrease in receptor expression for glycation products and NF-κB was noted in the hippocampus and brain cortical neurons after treatment with paeonol (at doses of 50 and 100 mg/kg). In addition, peonol significantly increased glutathione content and noticeably decreased nitric oxide synthase (iNOS) activity in hippocampal tissue [70]. In another study conducted on rats with streptozocin-induced diabetes, paeoniflorin (at doses of 5, 10 or 20 mg/kg) was shown to have a preventive effect against the onset of nephropathy [71]. Subsequent studies in rats with streptozocin-induced diabetes and Freud’s complete adjuvant showed that polysaccharide-2b present in *Moutan cortex* could significantly delay the onset and alleviate the degree of lens opacification in diabetic cataracts. Compared to the model group, the groups treated with polysaccharide-2b had reduced levels of malonylaldehyde (MDA), and, in the groups treated with its medium and high doses, reduced levels of glutathione peroxidase, superoxide dismutase and catalase were observed, as well as increased Na+/K+ ATP-ase activity. These results indicate a positive relationship between the dose of polysaccharide-2b and its effect [34].

Palbinone and certain triterpenoids isolated from *Moutan cortex* stimulated glucose uptake and glycogen synthesis via the AMPK pathway in a dose-dependent manner in human insulin-resistant HepG2 cells. These compounds may have significant potential in alleviating metabolic disorders associated with diabetic complaints [72] (Table 3).

### 5.7. Neuroprotective Effect

Studies demonstrated that *Moutan cortex* is effective in alleviating neuropathic pain in mice [19] and exhibits protective effects on neurons in a mouse model of 1-methyl-4-phenyl-1,2,3,6-tetrahydropyridine (MPTP)-induced Parkinson’s disease [57].

In studies conducted on nerve cell cultures, paeonol at concentrations of 12.5, 25 and 50 μmol/L was shown to protect rat neurons from oxygen and glucose deficiency-induced damage. As a result, it reduces morphological damage to cells and prolongs their life span. This effect may be related to inhibition of N-methyl-D-aspartate (NMDA) receptor binding capacity and reduction in intracellular calcium ion concentration [58]. Paeonol was also confirmed to suppress LPS-induced neuroinflammatory responses through suppression of the NF-κB pathway and mitogen-activated kinase (MAPK) [59]. In studies conducted on microglia cells, paeonol significantly inhibited the release of nitric oxide (NO) and the expression of iNOS and COX-2. Paeonol treatment also reduced ROS production and inhibited excessive ATP-induced cell migration. The anti-neuroinflammatory effect of paeonol was also found to be regulated by AMPK-α kinase and glycogen synthase kinase 3 α/β (GSK 3α/β) [60].

In addition, the protective properties of paeoniflorin at doses of 5 mg/kg administered twice daily for 14 days against ischemic brain injury in rats were confirmed by inhibiting the MAPKs/NF-κB-dependent inflammatory response [61]. It was also demonstrated that 1,2,3,4,6-penta-O-galloyl-β-D-glucopyranose can protect neuronal cells from oxidative stress by inducing the expression of the hemooxygenase-1 gene [62].

Additionally, resveratrol oligomers: resveratrol trimers-suffruticosol A, suffruticosol B, suffruticosol C, *trans*-suffruticosol D, *trans*-gnetin H and resveratrol dimer-trans-ε-viniferin, found in the seed coat extracts of *P. × suffruticosa*, had an effect on cholinesterase and the reduction in cytotoxicity induced by oxygen and glucose reoxygenation/reoxygenation (OGD/R) in PC12 cells (cell line that was derived from a transplantable rat pheochromocytoma) and on scopolamine-induced cognitive deficits in mice. The seed coat extracts of *P. × suffruticosa* display good inhibition of acetylcholinesterase and butyrylcholinesterase activities and significantly increase the viability of normal and OGD/R-injured PC12 cells. The seed coat extracts of *P. × suffruticosa* improve the cognitive performance of scopolamine-treated mice in behavioral tests. Furthermore, the seed coat extracts of *P. × suffruticosa* increase acetylcholinesterase, choline acetyltransferase, superoxide dismutase (SOD) and catalase (CAT) activities and acetylcholine, glutathione GSH and iterleukin-4 (IL-4) levels and decreases interleukin-1β (IL-1β), interleukin-6 (IL-6) and TNF-α levels in the model animals [63] (Table 3).

### 5.8. Effects in Neurodegenerative Diseases

Studies demonstrated that paeonol treatment can protect against many of the biochemical, morphological and behavioral changes resulting from amyloid-β administration in a rat model of Alzheimer’s disease. The results suggest that paeonol is a potential therapeutic agent in slowing down the pathogenic processes associated with the disease [22]. A study conducted on ICR mice (albino mice) in a D-galactose assay injected subcutaneously for 60 days at a dose of 50 mg/kg/day showed that paeonol at 50 and 100 mg/kg, together with D-galactose, increased acetylcholine and glutathione levels, restored superoxide dismutase activity and Na^+^/K^+^-ATPase levels, and decreased MDA levels and cholinesterase activity. In addition, paeonol alleviated neuronal damage in both the hippocampus and temporal cortex [110]. It was also confirmed that 1,2,3,4,6-penta-O-galloyl-β-D-glucopyranose inhibits the formation and destabilizes the pre-formation of amyloid-β fibrils in in vitro and in vivo models [111] (Table 3).

### 5.9. Hepatoprotective Effect

In in vivo studies, *Moutan cortex* extract was proven to have a protective effect against liver damage from paracetamol. The extract reduced glutathione deficiency and cytochrome P450 2E1 activity and protected against the destruction of hepatic DNA [73].

In another experiment, the effect of paeonol on model alcoholic liver damage in mice was studied. Paeonol treatment significantly reduced serum aminotransferase levels, liver cell damage, steatosis and inflammatory cell infiltration. In addition, paeonol significantly reduced hepatic mRNA expression of lipogenic genes and serum and tissue levels of inflammatory cytokines, tissue lipid peroxidation and neutrophil infiltration and inhibited hepatocyte apoptosis [74]. In addition, paeonol was shown to attenuate epirubicin-induced hepatotoxicity by inhibiting the PI3K/Akt/NF-κB pathway [75].

Paeoniflorin (at a dose of 50 mg/kg) injected into the tail vein in a mouse model protected against concanavalin A-induced liver inflammation by inhibiting certain pro-inflammatory cytokines, i.e., TNF-α, IL-6 and IFN-γ and down-regulating the NF-κB pathway [76]. Another study showed that paeoniflorin attenuated liver fibrosis by inhibiting hypoxia-inducible factor-1α, in part through the m-TOR-dependent pathway [77] (Table 3).

### 5.10. Anti-Allergic Effect

The anti-allergic effect of the ethanol extract of *Moutan cortex* was evaluated in some animal models. The raw material extract (at 30 and 100 mg/kg, i.p.) dose-dependently inhibited systemic anaphylactic shock induced by compound 48/80 (a polymer that increases histamine release) in mice. It also dose-dependently inhibited the scratching reflex induced by compound 48/80 or histamine at a dose of 100 mg/kg b.w. Increased vascular permeability induced by compound 48/80 or histamine was also inhibited by *Moutan cortex* extract. In addition, in vitro, the raw material reduced histamine release from rat peritoneal cell mast cells. Aiming to test the active component of the crude extract, it was suspended in water and extracted with ethyl acetate. Fractions insoluble in acetone extract (A) and soluble in it (B) were obtained. The effect of extract (B) was stronger than that of extract (A) in inhibiting histamine release. This study indicates that the raw material may be useful in alleviating symptoms of atopic dermatitis and other allergy-related diseases [112].

In vitro and in vivo studies showed that the ethanolic extract of *Moutan cortex* does not cause cytotoxicity in human mast cells. The ethanolic extract of the raw material (200 mg/kg) significantly inhibited the passive cutaneous anaphylactic reaction in vivo and inhibited the histamine release induced by compound 48/80 from rat peritoneal mast cells [113] (Table 3).

### 5.11. Immunomodulatory Activity

Two polysaccharides (PSAP-1 and PSAP-2) obtained from *P. × suffruticosa* flowers were tested for activity on RAW264.7 cells (macrophage cell line). The polysaccharides were subjected to chromatographic analysis, which showed that PSAP-1 was mainly composed of glucuronic acid, glucose, arabinose and galactose, while PASAP-2 was composed of rhamnose, galacturonic acid, glucose, arabinose and galactose. Research results showed that PSAP-1 and PSAP-2 can significantly stimulate Raw264.7 cell proliferation in a dose-dependent manner and further activate Raw264.7 cells by releasing immunoactive molecules such as nitric oxide, TNF-α and IL-6. In addition, PSAP-2 had higher immunomodulatory activity than PSAP-1 [114] (Table 3).

### 5.12. Antibacterial and Antifungal Effects

*P. × suffruticosa* buds extract showed the most efficient antibacterial effect against *Staphylococcus aureus* and *E. coli*, for which the minimum inhibition concentration (MIC) and minimum bactericide concentration (MBC) both were 1.56 mg/mL and 6.25 mg/mL, respectively [32,115].

Studies have shown that *Moutan cortex* has antifungal activity against Candida glabrata. The compound responsible for the above activity may be 1,2,3,4,6-penta-O-galloyl-β-D-glucopyranose, due to its cell wall degradation inducing activity [116] (Table 3).

**Table 3:** Directions and general mechanisms confirmed by scientific research of biological activity of *Paeonia × suffruticosa*.

Activity	Mechanism of Action	References
**Antioxidant activity**	Reduction in the production of reactive oxygen species (ROS) (ethanolic extract of *Moutan cortex*)	[45]
	Activation of the signaling of the transcription factor Nrf2, that controls the expression of genes encoding enzymes and cytoprotective proteins (paeonol)	[56,64]
**Cytoprotective activity**	Protection of thymocytes (“pre-T lymphocytes”) against oxidative damage caused by ^60^Co radiation (paeoniflorin)	[38]
	Protection of human cell lines (EA.hy926) against gamma-induced oxidative damage via the transcription factor pathway protection Nrf2/HO (paeoniflorin)	[39]
	Protection of the retinal pigment epithelium cells against oxidative stress, reduction in the production of ROS and inhibition of the activation of the caspase-3 pathway (paeoniflorin)	[40]
	Protection against damage and cell death caused by hydrogen peroxide in human HaCaT keratinocytes (galloilopaeoniflorin)	[41]
**Anti-inflammatory activity**	Inhibition of the expression of nitric oxide synthase (iNOS) and induced cyclooxygenase (COX-2) by suppressing the phosphorylation of the inhibitory protein (I-κBα), the transcription factor NF-κB (extract of *Moutan cortex*)	[20]
	Inhibition of the HMGB1-RAGE/TLR-2/TLR-4-NF-κB pathway (paeoniflorin)	[66]
	Inhibition of the synthesis of tumor necrosis factor (TNF-α) and the production of interleukin-6 (IL-6) in synoviocytes (cells of the synovial membrane of the joint capsule) (paeonol, paeoniflorin, 1,2,3,4,6-penta-O-galloyl- β-D-glucopyranose)	[67]
	Inhibition of the secretion of interleukin-8 (IL-8) and monocyte chemotactic protein in human monocytic cells (U937) (methanol extract from shoulder) regulating the production of TNF-α and interleukins-IL-1β, IL-6 and IL-10 by inactivating I-κBα (NF-κB inhibitory protein), extracellular signal regulated kinase (ERK1/2), N-kinases c-JUN terminal (JNK) and mitogen-activated protein kinases (p38 MAPK) (paeonol)	[69]
	Inhibition of the total expression of HMGB1, NF-κB P65 and TNF-α in the lung tissue of acute lung injury rats (paeonol)	[35]
	Suppressing the activity of M1 macrophage cells (pro-inflammatory) and increasing the function of M2 macrophage cells (anti-inflammatory) (paeoniflorin)	[36]
	Inhibition expressions of Il-6, TNF-α, NADPH oxidase 2 (NOX2), prostaglandin-endoperoxide synthase 2 (PTGS2), nucleobindin-2 (NUCB2)/nesfatin-1, intercellular adhesion molecule 1 (ICAM -1), vascular cell adhesion molecule 1 (VCAM-1), matrix metalloproteinase-3/13 (MMP-3/13) and degradation of type II collagen (paeonol)	[37]
**Anticancer activity**	Reduction resistance to paclitaxel in human breast cancer cells by regulating the expression of transgelin 2 (paeonol)	[42]
	Induction of an anti-tumor effect on colon cancer cells by suppressing prostaglandin synthesis (PGE-2) and expression of COX-2 (paeonol)	[43]
	Inhibition of the metastasis of melanoma and chondrosarcoma (paeonol)	[44]
	Induction of apoptosis of EMT6 breast cancer cells, HepG2 human liver cancer cells and HepA cells in a mouse model (paeonol)	[46,48]
	Inhibition of cell proliferation and induction of apoptosis on mouse gastric cancer cell lines (MFC) and on human gastric tumor cells SGC-7901 (paeonol)	[49]
	Decreases the expression of proteins regulating the release of cytochrome *c* from mitochondria (Bcl-2) and increased the expression of the apoptosis accelerating protein (Bax) in MFC and SGC-7901 (paeonol)	[49]
	Cells’ suppression of the signaling pathway for the gene encoding the single-pass Notch-1 transmembrane receptor in breast cancer cells (paeoniflorin)	[50]
	Inhibition of macrophage-dependent metastasis of lung cancer (paeoniflorin)	[50]
	Inhibition of proliferation and induction of apoptosis of human glioblastoma cells by up-regulation of microRNA-16 and down-regulation of metalloproteinase-9 (paeoniflorin)	[51]
	Reduction in proliferation on human SK-HEP-1 hepatocellular carcinoma cells (1,2,3,4,6-penta-O-galloyl-β-D-glucopyranose)	[52]
	Inhibition of the growth of bladder cancer cells (extract of *Moutan cortex*)	[53]
	Induction stress on endoplasmic reticulum (ER)-related proteostasis and affected mitochondrial membrane potential to increase autophagosome numbers and block their degradation (aqueous extracts of *Moutan cortex*)	[54]
	Inhibition of the proliferation of human liver cancer cell line (HepG2) and human hepatocarcinoma cell line (SMMC-7721) and induction apoptosis, concomitant with the down-regulation of survivin (paeonol)	[55]
**Cardioprotective and anti-atherosclerotic activity**	Inhibition of thrombocyte aggregation and blood coagulation (paeonol, paeoniflorin, benzoylpaeoniflorin and benzoyloxypaeoniflorin)	[21]
	Protective effect in the rat model of ischemia and reperfusion (extract of *Moutan cortex*)	[78]
	Inhibition of inflammatory processes and signaling pathways of iNOS (paeoniflorin)	[79]
	Reduction in vascular damage and the expression of E-selectin and intercellular adhesion molecule (ICAM-1) in a mouse model of the skin Arthus reaction (paeoniflorin)	[80]
	Increases thrombus recanalization by inducing endothelial growth factor-165 and up-regulating urokinase plasminogen activator (paeonol and paeoniflorin)	[81,82]
	Inhibition of the adhesion of monocytes, induced by the oxidized form of LDL, to the vascular endothelium by inhibiting the mitogen-activated kinase (MAPK) signaling pathway (paeonol)	[83]
	Prevention arterial thrombosis (paeoniflorin)	[84]
**Antidiabetic activity**	Inhibition of glucose uptake by intestinal brush border membrane vesicles (BBMV) and increased glucose uptake in human skin fibroblasts (Hs68) and Mouse adipocytes (3T3-L1) (extract of *Moutan cortex*)	[85]
	improved glucose tolerance (paeonol)	[85]
	Decrease in receptor expression for glycation products and NF-κB in the hippocampus and cortical neurons of the brain (paeonol)	[70]
	Increases the content of glutathione and noticeably reduces the activity of iNOS in the tissue of the hippocampus (paeonol)	[71]
	Delays the onset and alleviates the degree of lens opacities in diabetic cataracts (polysaccharide-2b present *Moutan cortex*)	[34]
	Stimulation of human insulin-resistant HepG2 cells glucose uptake and glycogen synthesis via the AMPK pathway (palbinon and some triterpenoids isolated from *Moutan cortex*)	[72]
**Neuroprotective activity**	Relieving neuropathic pain (*Moutan cortex* extract)	[19]
	Protective effect on neurons in a mouse model of Parkinson’s disease (*Moutan cortex* extract)	[57]
	Reduction in cell damage and extending cell viability by inhibiting the ability to bind the N-methyl-D-aspartate (NMDA) receptor and reducing the intracellular concentration of calcium ions (paeonol)	[59]
	Suppression of neuroinflammatory reactions by suppressing the NF-κB pathway and mitogen-activated kinases (MAPK) (paeonol)	[59]
	Inhibition of the release of NO and the expression of iNOS and COX-2 in microglia cells (paeonol)	[60]
	Regulation of AMPK-α kinase and glycogen synthase 3α/β kinase (GSK 3α/β) (paeonol)	[60]
	Protection of nerve cells against oxidative stress by inducing the expression of the hemoxygenase-1 gene (1,2,3,4,6-penta-O-galloyl-β-D-glucopyranose)	[62]
	Improves the cognitive performance of scopolamine-treated mice in behavioral tests (seed coat extracts of *P. × suffruticosa*)	[63]
	Increases acetylcholinesterase, choline acetyltransferase, superoxide dismutase (SOD) and catalase (CAT) activities and acetylcholine, glutathione (GSH) and IL-4 levels, and decreases IL-1β, IL-6 and TNF-α levels in a cell line that was derived from a transplantable rat pheochromocytoma (seed coat extracts of *P. × suffruticosa*)	[63]
**Activity in neurodegenerative diseases**	Increasing the level of acetylcholine and GSH (paeonol)	[22]
	Restoration of superoxide dismutase activity and concentration of Na +/K+-ATPase (paeonol)	[110]
	Reduction in MDA levels and cholinesterase activity (paeonol)	[110]
	Alleviated neuronal damage, both in the hippocampus and in the temporal cortex (paeonol)	[110]
	Inhibition of formation and destabilization of initial formation of amyloid-β fibrils in in vitro and in vivo models (1,2,3,4,6-penta-O-galloyl-β-D-glucopyranose)	[111]
**Hepatoprotective activity**	Reduction in GSH deficit, cytochrome P450 2E1 activity and protection against damage to hepatic DNA (extract of *Moutan cortex*)	[73,74,75,76,77]
	Decrease in serum transaminase levels, damage to liver cells, steatosis and infiltration of inflammatory cells (paeonol)	[74]
	Reduction in hepatic mRNA expression of lipogenic genes (paeonol)	[74]
	Lowering the level of inflammatory cytokines in serum and tissues, peroxidation of tissue lipids, neutrophil infiltration and inhibition of hepatocyte apoptosis (paeonol)	[75]
	Alleviation of liver fibrosis by inhibiting the hypoxia-1α-induced factor, partly by the m-TOR dependent pathway (paeoniflorin)	[76,77]
**Anti-allergic effect**	Inhibition of systemic anaphylactic shock (ethanol extract of *Moutan cortex*)	[112]
	Inhibition of the scratch reflex (ethanol extract of *Moutan cortex*)	[112]
	Inhibition of increased vascular permeability (ethanol extract of *Moutan cortex*)	[112]
	Reduction in histamine release from mast cells (ethanol extract of *Moutan cortex*)	[113]
**Immunomodulatory activity**	Stimulation of Raw264.7 (macrophage cell line) cell proliferation (polysaccharides obtained from *P. × suffruticosa* flowers)	[114]
	Activation of Raw264.7 cells by releasing immunoactive molecules such as NO, TNF-α and IL-6 (polysaccharides obtained from *P. × suffruticosa* flowers)	[114]
**Antibacterial and antifungal activity**	Inhibition of growth of Gram-positive bacteria: *Staphylococcus aureus* and Gram-negative bacteria: *Escherichia coli* (methanol extract of *Moutan cortex*)	[32,116]
	Inhibition of growth of *Candida glabrata* (1,2,3,4,6-penta-O-galloyl-β-D-glucopyranose)	[116]

## 6. Applications in Cosmetology

*Moutan cortex* extract also has scientifically proven cosmetic effects, such as antioxidant, anti-aging and skin brightening effects [86,87]. In studies on B16 cells used to study skin cancer, the extracts were shown to inhibit tyrosinase activity and 3,4-dihydroxyphenylalanine (DOPA), which contributes to the reduction in melanin content in cells [86]. Additionally, kinetic analyses revealed that the ethanol *Moutan cortex* extract and paeonol are noncompetitive tyrosinase inhibitors. The cellular melanin content and L-DOPA oxidation assays demonstrated that the ethanol *Moutan cortex* extract was an appropriate alternative whitening agent to paeonol and arbutin in ultraviolet-induced A2058 human melanoma cells. The ethanol *Moutan cortex* extract was also confirmed as a promising ingredient in sun protection and skin whitening cosmetics [87]. In vitro, *P. × suffruticosa* root extract and paeonol significantly inhibited UVB-induced phosphorylation of mitogen-activated protein kinase and activator protein 1 in keratinocytes, which consequently led to degradation of procollagen type I. In vivo, topical application of *P. × suffruticosa* root extract and paeonol attenuated UVB-induced matrix metalloproteinase-1 production and promoted procollagen type I in hairless mice [117]. Recent reports revealed paeonol from *P. × suffruticosa* exhibited good effects on chronic dermatitis, such as atopic dermatitis (AD) and psoriasis. One study analyzed the effects of paeonol on a mouse model of dry skin treated with acetone-ether-water (AEW). The results showed impressive activities in reducing scratching behavior and skin inflammation. The studies indicated that paeonol can ameliorate AEW-induced inflammatory response and itching behavior and reduce the expression of spinal astrocyte activity-dependent genes induced by AEW, reducing the expression of spinal astrocyte activity-dependent genes induced by AEW [88].

According to the CosIng (Cosmetic Ingredient Database) [118], in addition to extracts from the root and bark of *P. × suffruticosa*, it is also possible to use extracts from stems, leaves, flowers, from the whole plant, as well as from the biomass of callus cultures in the production of cosmetics in the countries of the European Union (Table 4) [118]. In cosmetics, hydrolates from flowers, roots and seed oil have also found application. These raw materials can be found mainly in facial skin care cosmetics with anti-aging, antioxidant, brightening and nourishing properties. In addition to the above-mentioned properties, hydrolats also give cosmetics a pleasant fragrance. Additionally, the *P. × suffruticosa* root is found in the filtrates of products obtained from the fermentation of the roots of various plant species by bacteria: *Acetobacter*, *Lactobacillus* and *Leuconostoc* and by fungi: *Aspergillus*, *Monascus* and *Saccharomyces* (Table 4). *Paeonia suffruticosa* root extract is most commonly used in cosmetic products. It is found in Korean (e.g., A’pieu, Holika Holika), French (e.g., L’Oreal, Yves Saint Laurent, Lancôme), Polish (e.g., Dermofuture) and American (e.g., Estée Lauder) cosmetics.

## 7. *Paeonia × suffruticosa*—Toxicity

*Moutan cortex* is a safe raw material. There are no studies confirming its toxic effect. Benzoic acid present in the extracts is considered a harmful component, but in this species, it is present at low levels [119]. Attention should be paid to the ease of contamination of the raw material with heavy metals familiar to soil, irrigation waters, atmospheric dust, car and industrial exhaust fumes, as well as pesticides and fertilizers [120]. *Moutan cortex* can become contaminated with exogenous substances such as heavy metals, pesticide residues or excessive sulfur content from sulfur fumigation. Therefore, the determination of trace elements in *Moutan cortex* is essential to ensure the high quality of the raw material. Due to these reasons, the places where this species is grown are important [121,122,123].

## 8. *Paeonia × suffruticosa*—Pant Biotechnological Studies

Increasingly, the species *P. × suffruticosa* is becoming the subject of biotechnology research due to breeding problems such as severe browning, difficulty in differentiation and rooting, and low regeneration efficiency. Establishing an efficient regeneration system is considered to be an important goal among peony researchers.

The first research on *P. × suffruticosa* in vitro cultures was carried out in 1977 by Gildow and Mitchell [124]. Tissue cultures of *P. × suffruticosa* were established using explants of etiolated stems. Callus formation was induced on agar-solidified Schenk and Hildebrandt medium (SH) containing the plant growth regulators 2,4-dichlorophenoxyacetic acid (2,4-D)—0.2 mg/L and kinetin (KIN)—0.1 mg/L. Growth was tested on a range of liquid media: SH/2, SH, SH × 2 and SH—M, containing 1250, 2500, 5000 and 2500 mg/L potassium nitrate. The SH—M medium, additionally, contained 1650 mg/L ammonium nitrate. Growth measured as increased fresh weight was best on the SH/2, SH and SH—M media and was curtailed on the SH × 2 medium [124].

Zhu et al. recently described the callus induction, shoot organogenesis and plant regeneration using young *P. × suffruticosa* leaves as explants. Various media containing diverse plant growth regulators were assessed for their potency in propagation. After exposure of dark-adapted leaf discs to 30 μmol/m^2^s of light, inoculation in a Murashige and Skoog (MS) medium containing 0.2 mg/L 2,4-D, 0.2 mg/L 1-naphthaleneacetic acid (NAA) and 3.0 mg/L thidiazuron (TDZ) resulted in the highest callus induction rate, with values reaching up to 87.8%. The studies also documented MS with 0.2 mg/L NAA, 2.0 mg/L, 6-benzyladenine (6-BA) and 2.0 mg/L KIN to be the optimal medium for further callus proliferation under light. Inoculation on MS containing 2.0 mg/L 6-BA, 0.2 mg/L NAA and 0.3 mg/L TDZ medium allowed callus cultures to differentiate into adventitious shoots, whereas a similar rate of root formation was detected when 1/2 MS containing 0.1 mg/L NAA, 0.05 mg/L 3-indolebutyric acid (IBA) and 30 g/L sucrose medium was used [125].

Protocol for high-frequency callus induction and establishment of *P. × suffruticosa* was described by Chen et al. [126]. Cultures were started from flower petals as explants. MS medium supplemented with 2.0 mg/L 2,4-D, 1.5 mg/L 6-BA and 0.3 mg/L NAA was identified as the best medium for callus induction, achieving an induction rate of up to 98.52%. The highest *P. × suffruticosa* proliferation rate (234%) was achieved on MS medium supplemented with 0.2 mg/L NAA and 3.0 mg/L 6-BA. The highest callus differentiation rate (34.81%) was achieved on MS supplemented with 2.0 mg/L 6-BA and 0.5 mg/L zeatin (Zea). The highest rooting rate was 23.33% when using 1/2 MS supplemented with 0.1 mg/L NAA and 0.05 mg/L IBA [126].

## 9. Conclusions

*Paeonia × suffruticosa* root bark, under the name of *Moutan cortex*, was introduced for official medicinal use in European Union countries by Supplement 9.4 to the European Pharmacopoeia in 2018 [23]. This plant has long been known and used in TCM. According to the indications of TCM, the raw material has an antipyretic effect, regulates hormonal cycles in women, improves blood circulation, reduces swelling and accelerates the treatment of ulcers. Contemporary professional pharmacological research of this raw material has proven numerous valuable directions of its activity, e.g., antioxidant, cytoprotective, anti-cancer, immunomodulating, anti-inflammatory, cardioprotective, anti-atherosclerotic, anti-diabetic, hepatoprotective as well as antimicrobial properties. Moreover, the raw material shows neuroprotective activity and can be used in neurodegenerative diseases. Mainly two compounds present in the raw material have been indicated as responsible for this wide range of activity directions—paeonol (phenolic compound) and paeoniflorin (monoterpenoid glycoside), and, partly, also 1,2,3,4,6-penta-O-galloyl-β-D-glucopyranose. The roots, bark of the roots and other organs of *P. × suffruticosa*, hydrolates and seed oil, as well as callus cultures, can be used in accordance with the CosIng base in the European Union countries in the production of cosmetics.

There has been some biotechnological research aimed at developing effective in vitro micropropagation protocols of *P. × suffruticosa*.

## Figures and Tables

**Table 2:** The chemical composition of *Paeonia × suffruticosa* flowers.

Group of Compounds	Compounds	References
**Monoterpenoids**	3-carene, citronellal, citronellol, citronellyl acetate, nerol, allo-ocimene, β-ocimene, (E)-β-ocimene, (Z)-β-ocimene, O-cymene, β-cymene, (E)- geraniol, (Z)- geraniol, *cis*-linalool oxide, linalool, *trans*-linalool oxide (furanoid) β-myrcene, α-phellandrene, α-pinene, (1s)-(-)-β-pinene, α-terpinolene, α-terpineol	[105,106]
**Monoterpenoid glycosides**	benzoylpaeoniflorin, benzoyloxypaeoniflorin, debenzoylgalloylpaeoniflorin, 8-debenzoylpaeoniflorin, galloyloxypaeoniflorin, galloylpaeoniflorin, oxypaeoniflorin, paeoniflorin, paeonolide	[107]
**Phenols and their derivatives**	mudanoside A, mudanpioside C, mudanpioside E, mudanpioside H, mudanpioside J, paeonol	[107]
**Sesquiterpenoids**	β-cadinene, *cis*-calamenene, caryophyllene, β-copaene, α-cubebene, α-farnesene, germacrene D, γ-muurolene	[105,106]
**Alcohols**	1-decanol, 2-ethyl hexanol, *cis*-3-hexen-1-ol, 1-nonanol, *cis*-3-nonen-1-ol	[105,106]
**Alkanes**	1-chloro-octadecane, cyclotetradecane, dodecane, eicosane, heptadecane, hexadecane, 8-hexylpentadecane, nonane, octane, tetradecane, undecane	[105,106]
**Alkenes**	1-pentadecene	[105,106]
**Alkatrienes**	3,4-dimethyl-2,4,6-octatriene, (E)-4,8-dimethylnona-1,3,7-triene, 1,3,8-triene-*p*-menthatriene, 1,5,8-triene-*p*-menthatriene	[105,106]
**Adehydes**	benzeneacetaldehyde, decanal, geranial, heptanal, myrtanal, neral, octanal	[106]
**Phenolic acids**	gallic acid, gallic acid-di-O-glucoside, gentisic acid-5-O-glucoside, *p*-hydroxybenzoic acid	[107]
**Tannins**	glucogallin	[107]
**Flavonoids**	apigenin, apigenin-7-O-glucoside, chrysoeriol, 6,3′-dimethoxyquercetin-di-O-glucoside, diosmin, eriodictyol-7-O-glucoside, hederagenin, isorhamnetin-3-O-glucoside, isorhamnetin-7-O-glucoside, isorhamnetin-3,7-di-O-glucoside, kaempferol-3-O-(2′’-O-galloyl)-glucoside, kaempferol-3-O-glucoside, kaempferol-3-O-rutinoside, kaempferol-3,7-di-O-glucoside, luteolin, monoxerutin, patuletin-3,5-di-O-glucoside, rhoifolin, quercetin-3-O-glucoside, quercetin-O-di-glucoside	[107]
**Iridoid glycosides**	geniposide, loganin, morroniside	[107]
**Furans**	2-pentylfuran	[106]
**Aminoacids**	leucine, tryptophan	[107]
**Sugars**	sucrose	[107]

**Table 4:** Possible applications of *Paeonia × suffruticosa* in cosmetology as recommended by the CosIng database.

INCI Name	Description	Functions
*Paeonia × suffruticosa* bark extract	The extract of the bark of the Chinese Peony, *Paeonia × suffruticosa, Paeoniaceae.*	Skin conditioning
*Paeonia × suffruticosa* branch/flower/leaf extract	The extract of the branches, flowers and leaves of the Chinese Peony, *Paeonia × suffruticosa*, *Paeoniaceae.*	Skin conditioning
*Paeonia × suffruticosa* callus extract	The extract of the callus of *Paeonia × suffruticosa*, *Paeoniaceae*.	Antimicrobial Antioxidant Hair conditioning Skin protecting
*Paeonia × suffruticosa* extract	The extract of the whole plant *Paeonia × suffruticosa*, *Paeoniaceae*.	Skin conditioning
*Paeonia × suffruticosa* flower extract	The extract of the flowers of *Paeonia × suffruticosa*, *Paeoniaceae*.	Skin conditioning
*Paeonia × suffruticosa* flower water	The aqueous solution of the steam distillate obtained from the flowers of the Chinese Peony, *Paeonia × suffruticosa*, *Paeoniaceae*.	Fragrance
*Paeonia × suffruticosa* phytoplacenta extract	The extract of the phytoplacenta cells directly isolated from *Paeonia × suffruticosa* or grown in culture, *Paeoniaceae*.	Antimicrobial Antioxidant Hair conditioning Skin conditioning
*Paeonia × suffruticosa* root extract	The extract of the roots of the Chinese Peony, *Paeonia × suffruticosa, Paeoniaceae*.	Skin protecting
*Paeonia × suffruticosa* root water	The aqueous solution of the steam distillates obtained from the roots of the Chinese Peony, *Paeonia × suffruticosa*, *Paeoniaceae.*	Flavoring Fragrance Perfuming Skin conditioning
*Paeonia × suffruticosa* seed oil	The oil expressed from the seeds of *Paeonia × suffruticosa*, *Paeoniaceae*.	Skin conditioning Skin conditioning-emollient
*Aspergillus*/*Paeonia × suffruticosa* bark/Honeysuckle flower/*Forsythia* fruit (*Astragalus membranaceus*/*Gentiana scabra*/*Licorice*/*Rehmannia glutinosa*/*Rheum palmatum*/*Scrophularia buergeriana*) root/(castor/rice) seed ferment extract	The extract of the product obtained by the fermentation of the bark of *Paeonia × suffruticosa*; the flower of *Lonicera japonica* (honeysuckle); the fruit of *Forsythia suspensa* (forsythia); the roots of *Astragalus membranaceus*, *Gentiana scabra*, *Glycyrrhiza glabra* (licorice), *Rehmannia glutinosa* (rehmannia), *Rheum palmatum* and *Scrophularia buergeriana*; the seeds of *Oryza sativa* (rice), and *Ricinus communis* (castor), by the microorganism *Aspergillus*.	Skin conditioning
*Lactobacillus*/(*Achyranthes bidentata*/*Angelica gigas*/*Angelica pubescens*/*Angelica tenuissima*/*Asarum sieboldi*/*Cnidium officinale*/*Ledebouriella divaricata*/*Paeonia × suffruticosa*) Root/*Eucommia ulmoides* Bark/*Magnolia liliflora* Bud Ferment Filtrate	The filtrate of the product obtained by the fermentation of the roots of *Achyranthes bidentata*, *Angelica gigas*, *Angelica pubescens*, *Angelica tenuissima*, *Asarum sieboldi*, *Cnidium officinale*, *Ledebouriella divaricata*, *Paeonia × suffruticosa*, the bark of *Eucommia ulmoides* and the buds of *Magnolia liliflora* by the microorganism, *Lactobacillus*.	Skin conditioning-miscellaneous
*Lactobacillus*/(*Cudrania tricuspidata*/*Paeonia × suffruticosa*) Bark/*Lycium chinense* Fruit/Apricot Kernel/*Artemisia capillaris* Leaf/(*Angelica dahurica*/*Scutellaria baicalensis*) Root/Soybean Seed/*Houttuynia cordata*/Mistletoe/*Poria Cocos* ferment filtrate	The filtrate of the product obtained by the fermentation of the bark of *Cudrania tricuspidata*, and *Paeonia × suffruticosa*; the fruits of *Lycium chinense*; the kernels of *Prunus armeniaca* (apricot); the leaves of *Artemisia capillaris*; the roots of *Angelica dahurica*, and *Scutellaria baicalensis*; the seeds of *Glycine soja* (soybean); the whole plants, *Houttuynia cordata*, and *Viscum album* (mistletoe); and the fungus, *Poria cocos*, by the microorganism *Lactobacillus.*	Humectant Skin conditioning
*Lactobacillus*/*Ledebouriella divaricate* root/*Angelica dahurica* root/*Angelica tenuissima* root/*Magnolia liliflora* bud/*Asarum sieboldin* root/*Paeonia × suffruticosa* root/*Cnidium officinale* root/*Angelica gigas* root/*Aukcklandia lappa* root/*Achyranthes japonica* root/*Eucommia ulmoides* bark/*Angelica pubescens* root/*Aconitum koreanum* root ferment filtrate	The a filtrate of the product obtained by the fermentation of the roots of *Ledebouriella divaricata*, *Angelica dahurica*, *Angelica tenuissima*, *Asarum sieboldi*, *Paeonia × suffruticosa*, *Cnidium officinale*, *Angelica gigas*, *Auklandia lappa*, *Achyranthes japonica*, *Angelica pubescens*, and *Aconitum koreanum*, the buds of *Magnolia liliflora* and the bark of *Eucommia ulmoides* by the microorganism, *Lactobacillus.*	Skin conditioning
*Leuconostoc*/*Ledebouriella divaricata* root/*Angelica dahurica* root/*Angelica tenuissima* root/*Magnolia liliflora* bud/*Asarum sieboldi* root/*Paeonia × suffruticosa* root/*Cnidium officinale* root/*Angelica gigas* root/*Aucklandia lappa* root/*Achyranthes japonoica* root/*Eucommia ulmoides* bark/*Angelica pubescens* root/*Aconitum koreanum* root ferment	The product obtained by the fermentation of the roots of *Ledebouriella divaricata*, *Angelica dahurica*, *Angelica tenuissima*, *Asarum sieboldi*, *Paeonia × suffruticosa*, *Cnidium officinale*, *Angelica gigas*, *Aucklandia lappa*, *Achyranthes japonoica*, *Angelica pubescens*, *Aconitum koreanum*, the buds of *Magnolia liliflora*, and the bark of *Eucommia ulmoides*, by the microorganism, *Leuconostoc*.	Skin Conditioning
*Monascus*/*Paeonia × suffruticosa* flower/rice bran ferment filtrate	The filtrate of the product obtained by the fermentation of the flowers of *Paeonia × suffruticosa* and the bran of *Oryza sativa* (rice) by the microorganism, *Monascus*.	Anti-sebum Skin conditioning
*Acetobacter*/*Ledebouriella divaricata* root/*Angelica dahurica* root/*Angelica tenuissama* root/*Magnolia liliflora* bud/*Asarum sieboldi* root/*Paeonia × suffruticosa* root/*Cnidium officinale* root/*Angelica gigas* root/*Aucklandia lappa* root/*Achyranthes japonica* root/*Eucommia ulmoides* bark/*Angelica pubescens* root/*Aconitum koreanum* root ferment filtrate	The filtrate of the product obtained by the fermentation of *Ledebouriella divaricata* roots, *Angelica dahurica* roots, *Angelica tenuissama* roots, *Magnolia liliflora* buds, *Asarum sieboldi* roots, *Paeonia × suffruticosa* roots, *Cnidium officinale* roots, *Angelica gigas* roots, *Auklandia lappa* roots, *Achyranthes japonica* roots, *Eucommia ulmoides* bark, *Angelica pubescens* roots, *Aconitum koreanum* roots by the microorganism *Acetobacter.*	Skin conditioning
*Saccharomyces*/(*Achyranthes bidentata*/*Angelica gigas*/*Angelica pubescens*/*Angelica tenuissima*/*Asarum sieboldi*/*Cnidium officinale*/*Ledebouriella divaricata*/*Paeonia × suffruticosa*) root/*Eucommia ulmoides* bark/*Magnolia liliflora* bud ferment filtrate	The filtrate of the product obtained by the fermentation of the roots of *Achyranthes bidentata*, *Angelica gigas*, *Angelica pubescens*, *Angelica tenuissima*, *Asarum sieboldi*, *Cnidium officinale*, *Ledebouriella divaricata*, *Paeonia × suffruticosa*, the bark of *Eucommia ulmoides* and the buds of *Magnolia liliflora* by the microorganism, *Saccharomyces*.	Skin conditioning
*Saccharomyces*/*Achyranthes bidentat* root/*Angelica gigas* root/*Angelica pubescens* root/*Angelica tenuissima* root/*Asarum sieboldi* root/*Cnidium officinale* root/*Eucommia ulmoides* bark/*Ledebouriella divaricata* root/*Magnolia liliflora* bud/*Paeonia × suffruticosa* root ferment filtrate	The filtrate of the product obtained by the fermentation of the roots of *Achyranthes bidentata*, *Angelica gigas*, *Angelica pubescens*, *Angelica tenuissima*, *Asarum sieboldi*, *Cnidium officinale*, *Ledebouriella divaricata*, *Paeonia × suffruticosa*, the buds of *Magnolia liliflora*, and the bark of *Eucommia ulmoides* by the microorganism, *Saccharomyces*.	Skin conditioning
*Saccharomyces*/*Achyranthes bidentata* root/*Angelica gigas* root/*Angelica pubescens* root/*Angelica tenuissima* root/*Cnidium officinale* root/*Eucommia ulmoides* bark/*Ledebouriella divaricata* root/*Paeonia × suffruticosa* root ferment filtrate	The filtrate of the product obtained by the fermentation of the roots of *Achyranthes bidentata*, *Angelica gigas*, *Angelica pubescens*, *Angelica tenuissima*, *Cnidium officinale*, *Ledebouriella divaricata*, *Paeonia × suffruticosa*, and the bark of *Eucommia ulmoides* by the microorganism, *Saccharomyces*.	Skin conditioning
*Saccharomyces*/*Camellia japonica* flower/*Castanea crenata* shell/*Diospyros kaki* leaf/*Paeonia × suffruticosa* root/*Rhus javanica*/*Sanguisorba officinalis* root extract ferment filtrate	The filtrate of the product obtained by the fermentation of *Camellia japonica* flower extract, *Castanea crenata* shell extract, *Diospyros kaki* leaf extract, *Paeonia × suffruticosa* root extract, *Rhus javanica* extract and *Sanguisorba officinalis* root extract by the microorganism, *Saccharomyces*.	Skin conditioning
*Saccharomyces*/*Cyperus rotundus* root/*Magnolia obovata* bark/*Paeonia × suffruticosa* root/*Peach kernel* ferment extract filtrate	The filtrate of the extract of the product obtained by the fermentation of the roots of *Cyperus rotundus* and *Paeonia × suffruticosa*, the bark of *Magnolia obovata*, and the kernel of *Prunus persica* (peach) by the microorganism, *Saccharomyces*.	Skin conditioning
*Saccharomyces*/*Ledebouriella divaricata* root/*Achyranthes bidentata* root/*Cnidium officinale* root/*Eucommia ulmoides* bark/*Angelica gigas* root/*Paeonia × suffruticosa* root/*Angelica tenuissim* root/*Asarum sieboldi* root/*Angelica pubescens* root/*Magnolia liliflora* bud ferment filtrate	The filtrate of the product obtained by the fermentation of the roots *of Ledebouriella divaricata*, *Achyranthes bidentata*, *Cnidium officinale*, *Angelica gigas*, *Paeonia × suffruticosa*, *Angelica tenuissima*, *Asarum sieboldi*, *Angelica pubescens*, the bark of *Eucommia ulmoides* and the buds of *Magnolia liliflora* by the microorganism, *Saccharomyces.*	Skin conditioning
